# Atypical presentation of an oesophageal carcinoma with metastases to the left buttock: a case report

**DOI:** 10.1186/1757-1626-2-6691

**Published:** 2009-05-18

**Authors:** Sarah Smyth, Mark E O'Donnell, Susim Kumar, Atiq Hussain, Brian Cranley

**Affiliations:** 1 Department of General Surgery, Daisy Hill HospitalNewry BT35 8DRNorthern Ireland; 2 Faculty of Life and Health Sciences, University of UlsterJordanstown Campus, Shore Rd, Newtownabbey BT37 0QBNorthern Ireland

## Abstract

**Introduction:**

Oesophageal carcinomas represent 3% of all cancers in the UK accounting for 7650 new cases per annum. Oesophageal cancer may be associated with swallowing abnormalities, localised mass pressure effects, lymphatic or distant metastatic spread.

**Case presentation:**

We report a 50-year-old man who presented with a painful, enlarging, solid, fixed lesion adjacent to the left buttock with associated dysphagia. Initial endoscopic assessment suggested severe oesophageal inflammation while the lesion in the buttock area was presumed to be a primary soft-tissue neoplasm. However, subsequent histological assessment confirmed a primary oesophageal squamous carcinoma with metastatic spread to the buttock.

**Conclusion:**

We discuss the clinical presentation, investigative modalities, and current therapeutic guidelines associated with this rare metastasis and present other atypical oesophageal musculoskeletal metastases. We emphasise the need to consider all aspects of patient symptomatology during the investigation of any atypical lesion.

## Introduction

Oesophageal carcinomas represent 3% of all cancers in the UK accounting for 7650 new cases per annum [1]. Oesophageal cancer may be associated with swallowing abnormalities, localised mass pressure effects, lymphatic or distant metastatic spread [1].

## Case presentation

A 50-year-old male Caucasian patient from Northern Ireland presented with a 6-month history of an enlarging firm tender swelling adjacent to his left buttock. He also described progressive dysphagia to solids with intermittent odynophagia. He had associated anorexia and weight loss of 7 kilograms over a 3-month period. He had no other gastrointestinal symptomatology. He had no other significant medical history but smoked 40 cigarettes a day. There was no history of oesophageal cancer but his father died from colon cancer aged 87. He drank alcohol occasionally and had no previous proton pump inhibitor usage.

On examination, he was haemodynamically stable. Abdominal examination was unremarkable. However, he had a suspicious 18 cm × 12 cm mass arising adjacent to his left buttock that was tender on palpation and fixed to the underlying left sacroiliac joint. Initial haematological investigations demonstrated a haemoglobin level of 16.9 g/dl and a white cell count of 9.71 × 10^9^/litre. Urea and electrolytes were normal. The alkaline phosphatase was 129 μ/L and corrected calcium was 3.07 mmol/L. Both carcinoembyronic antigen and CA 19-9 tumour marker levels were raised at 18.7 (normal range = 0-4) and 235 (normal range = 0-37) units/ml respectively.

**Figure 1.**
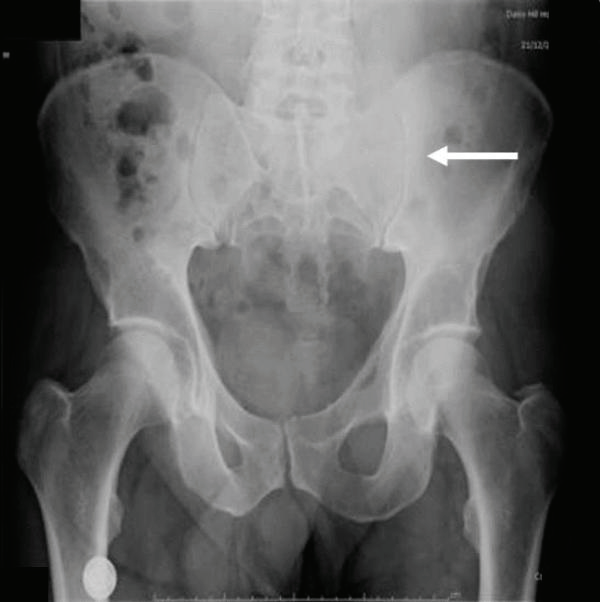
There is increased soft tissue density over the left sacro-iliac joint (white arrow). No definite bone destruction can be visualised on this plain film X-ray.

**Figure 2.**
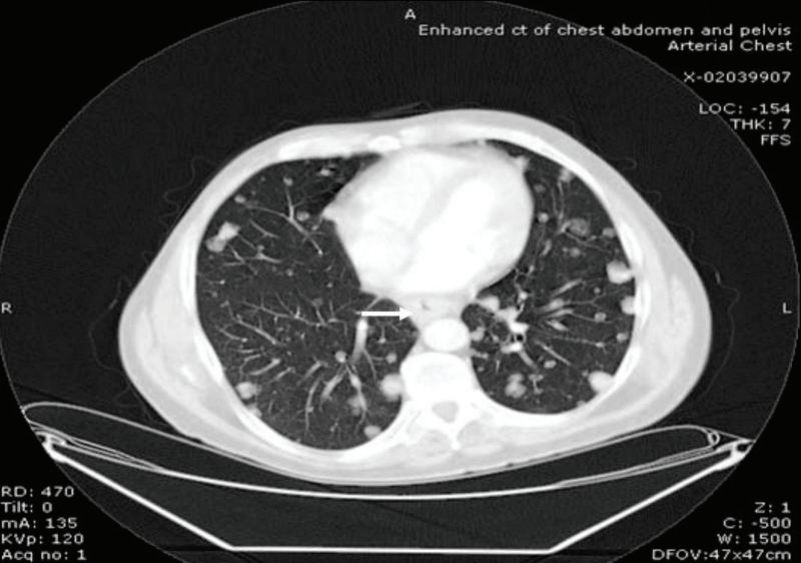
This section of CT shows evidence of the multiple opacifications in both hemithoraces in keeping with likely metastatic deposits. There is also a good illustration of the thick walled oesophagus at the level of the posterior mediastinum (white arrow). This was in keeping with a neoplastic growth of the oesophagus.

**Figure 3.**
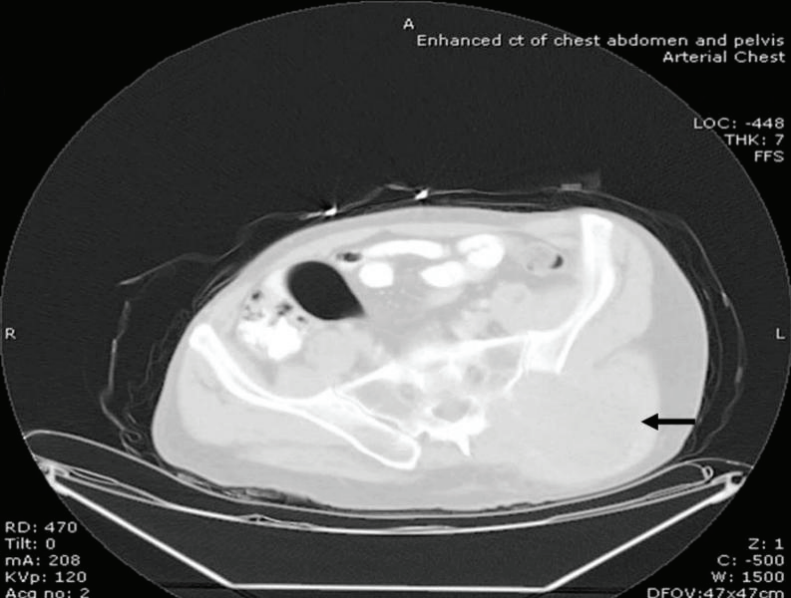
CT scan of large soft tissue mass in the posterior sacro-iliac joints (black arrow).

A plain X-ray of the pelvis demonstrated a soft tissue density over the left sacroiliac joint without any evidence of definite bone destruction (Figure 1). An urgent OGD revealed marked inflammatory changes in the distal oesophagus at 35 cm with a slightly raised mucosa which was suspicious of an underlying malignancy. Multiple biopsies were taken. A subsequent CLO-test was positive for helicobacter pylori and he was commenced on appropriate eradication therapy combined with oral omeprazole 20 mg twice a day. A contrast-enhanced computerised tomography of the chest, abdomen and pelvis revealed multiple opacities in the left and right hemithoraces suspicious of metastatic deposits (Figure 2). The oesophagus was thick walled at the level of the posterior mediastinum in keeping with a possible neoplastic lesion. A large soft tissue mass was identified arising from the posterior sacro- iliac joints (Figure 3). There was erosion of the iliac bone into the sacro-iliac joint with increased vascularity to the left gluteal musculature. The differential diagnosis included an oesophageal lesion or a primary bone tumour such as a chondrosarcoma or osteosarcoma which had metastasised to the lung or even a dual pathology. Although not usually associated with such widespread metastatic disease, a solitary plasmacytoma was also considered. Histopathological assessments from the oesophagus confirmed a squamous carcinoma while an ultrasound guided biopsy from the left gluteal mass revealed the presence of a metastatic squamous cell carcinoma originating from the primary oesophageal tumour.

Unfortunately with the advanced nature of the patient's neoplastic disease, aggressive surgical intervention was not appropriate. Oesophageal stenting was discussed with the patient and delayed due to a lack of complete obstructive symptomatology. He had a 2-week course of palliative radiotherapy (10 fraction course) to the posterior aspect of his left pelvis with a moderate response. Unfortunately he has developed a left foot drop which may be related to possible metastatic sacral nerve involvement. Although palliative chemotherapy was planned, he was not fit to undergo the treatment and was referred to the palliative care team. His prognosis remains guarded.

## Discussion

Oesophageal cancer occurs in 3% of the population in the UK. In northern China and Iran, it exceeds 100 per 100,000 individuals. In America, the incidence is less than 5 per 100,000, although rates are nearly quadruple for African Americans. The commonest site of oesophageal cancer is the lower third of the oesophagus, followed by the upper and middle thirds. The Scottish Audit of Gastric and Oesophageal Cancer found that adenocarcinoma of the oesophagus was more frequent than squamous cell carcinoma (SCC) in a ratio of 5:4 [1]. Oesophageal cancer is more common over the age of 55 years (median age 72). Male sex, smoking and alcohol are risk factors for development of SCC of the oesophagus while Barrett's oesophagus predisposes to adenocarcinoma [1]. Tylosis, pernicious anaemia, achalasia and coeliac disease are all associated with a small but increased risk of squamous cell carcinoma [2].

Predominant symptomatology includes dyspepsia and progressive dysphagia. Other symptoms include anorexia, weight loss, recurrent vomiting or gastrointestinal haemorrhage [3]. Barium swallow or endoscopic assessment (OGD) are first line investigative modalities while endoscopic ultrasound and computerised tomography permit disease staging [4]. Bronchoscopy though not routinely advocated, is useful in revealing tracheobronchial invasion, especially with upper and middle third lesions. There is evidence that PET scanning is slightly more sensitive and specific in the detection of distant metastasis compared to CT but not for local lymph node detection [5].

Our patient complained of a painful enlarging buttock mass which was assessed and biopsied radiologically. Although oesophageal adenocarcinoma metastasising to the gluteus minimus has been previously reported, this is the first case of SCC metastasising to the buttock [4]. Other atypical SCC oesophageal metastases include the iris while metastasis to the buttock from carcinomas involving the urinary bladder, kidneys and larynx have been documented [5]. Oesophageal adenocarcinomas have also been reported to metastasise to rare bony areas such as the mandible [6].

The prognosis of oesophageal cancer is poor with the majority of patients with an unresected primary surviving less than 6 months. A study of 838 patients with oesophageal tumours revealed that approximately 18% had metastases at diagnosis [7]. Five year survival is greater than 80% for mucosal lesions, 50-80% for submucosal infiltration, and 20% with more advanced disease [8]. Radical surgery is recommended for systemically fit patients with localised T1 and T2 tumours. Palliative radiotherapy can improve dysphagia in 50-85% of patients whilst providing symptomatic relief from distant metastases as shown in this case [9]. Laser therapy or stent insertion are usually reserved for more severe dysphagia.

## List of abbreviations

CLO test: Camplyobacter-like organism test
CT: Computerized tomography
OGD: Oesophagogastroduodenoscopy
SCC: Squamous cell carcinoma

## References

[bib-001] Clinical Resource and Audit Group (CRAG) (2002). Scottish Audit of Gastric and Oesophageal Cancer: Report 1997-2000.  http://www.crag.scot.nhs.uk/committees/ceps/reports/0_prelims.pdf .

[bib-002] Sandler RS, Nyrén O, Ekbom A, Eisen GM, Yuen J, Josefsson S (1995). The risk of esophageal cancer in patients with achalasia. A population-based study. JAMA.

[bib-003] Scottish Intercollegiate Guidelines Network (2003). Dyspepsia. http://www.sign.ac.uk/guidelines/fulltext/68/section1.html.

[bib-004] Kelly S, Harris KM, Berry E, Hutton J, Roderick P, Cullingworth J, Gathercole L, Smith MA (2001). A systematic review of the staging performance of endoscopic ultrasound in gastro-oesophageal carcinoma. Gut.

[bib-005] Liberale G, Van Laethem JL, Gay F, Goldman S, Nagy N, Coppens E, Gelin M, El Nakadi I (2004). The role of PET scan in the preoperative management of oesophageal cancer. Eur J Surg Oncol.

[bib-006] Tamiolakis D, Tsamis I, Thomaidis V, Lambropouolu M, Alexiadis G, Venizelos I, Jivanakis T, Papadopoulos N (2007). Jaw Bone Metastasis: Four cases. Acta Dermatovenerol Alp Panonica Adriat.

[bib-007] Quint LE, Hepburn LM, Francis IR, Whyte RI, Orringer MB (1995). Incidence and distribution of distant metastases from newly diagnosed esophageal carcinoma. Cancer.

[bib-008] Hölscher AH, Bollschweiler E, Schneider PM, Siewert JR (1997). Early adenocarcinoma in Barrett's oesophagus. Br J Surg.

[bib-009] Albertsson M, Ewers SB, Widmark H, Hambraeus G, Lillo-Gil R, Ranstam J (1989). Evaluation of the palliative effect of radiotherapy for esophageal carcinoma. Acta Oncol.

